# Role of Computational Methods in Going beyond X-ray Crystallography to Explore Protein Structure and Dynamics

**DOI:** 10.3390/ijms19113401

**Published:** 2018-10-30

**Authors:** Ashutosh Srivastava, Tetsuro Nagai, Arpita Srivastava, Osamu Miyashita, Florence Tama

**Affiliations:** 1 Institute of Transformative Bio-Molecules (WPI), Nagoya University, Nagoya, Aichi 464-8601, Japan; srivastava.ashutosh@c.mbox.nagoya-u.ac.jp; 2 Department of Physics, Graduate School of Science, Nagoya University, Nagoya, Aichi 464-8602, Japan; tnagai@nagoya-u.jp (T.N.); srivastava.arpita@g.mbox.nagoya-u.ac.jp (A.S.); 3 RIKEN-Center for Computational Science, Kobe, Hyogo 650-0047, Japan

**Keywords:** hybrid modeling, integrative modeling, molecular dynamics, X-ray crystallography

## Abstract

Protein structural biology came a long way since the determination of the first three-dimensional structure of myoglobin about six decades ago. Across this period, X-ray crystallography was the most important experimental method for gaining atomic-resolution insight into protein structures. However, as the role of dynamics gained importance in the function of proteins, the limitations of X-ray crystallography in not being able to capture dynamics came to the forefront. Computational methods proved to be immensely successful in understanding protein dynamics in solution, and they continue to improve in terms of both the scale and the types of systems that can be studied. In this review, we briefly discuss the limitations of X-ray crystallography in studying protein dynamics, and then provide an overview of different computational methods that are instrumental in understanding the dynamics of proteins and biomacromolecular complexes.

## 1. Introduction

Ever since the first protein structure was solved sixty years ago [1], X-ray crystallography has been the most important experimental method for understanding protein structure and relating it to its function. Out of over 140,000 structures in the Protein Data Bank (PDB), about 90% were determined using X-ray crystallography. These structures contributed to shedding light on many important biological phenomena by providing mechanistic insight into the functions of the proteins involved in biological processes [2]. The protein structures determined by X-ray crystallography were responsible for understanding the basic mechanisms involved in enzyme function, cellular signaling, cellular recognition, etc., as well as for the development of pharmaceutical interventions to modulate these mechanisms via structure-based drug discovery [2].

The structures determined by X-ray crystallography represent an averaged conformation. However, living systems are dynamic and so are their components including proteins. Consequently, the protein structure–function paradigm, entailing the intimate relationship between the three-dimensional structure and function of proteins was expanded to the protein structure–function–dynamics paradigm. As per recent studies [3,4], the role of protein structural dynamics seems not only limited to the function, but also to the evolution of function, making it crucial to take dynamics into consideration whilst trying to understand biological processes. Limitations of crystallography for providing insight into the conformational dynamics of biological macromolecules can be complemented by several experimental methods for studying solution dynamics. These include nuclear magnetic resonance (NMR) spectroscopy, small-angle X-ray scattering (SAXS), hydrogen exchange mass spectrometry (HX-MS), and recently, cryo-electron microscopy. However, these approaches have their own limitations, particularly with respect to the applicable systems, resolutions, etc. In this regard, computational methods proved to be crucial for studying the dynamics of biomacromolecules, particularly at shorter time scales on the order of nanoseconds to microseconds. With recent advances in supercomputer architecture, graphics processing unit (GPU)-based computing, and faster algorithms, microsecond- to millisecond-scale studies of large systems are becoming routine and, with the current pace, studying second-scale dynamics may be possible in the next few years [5]. In this review, we focus on how computational methods contributed to mitigating the limitations of biomolecular crystallography.

We begin with brief descriptions of some of the most pressing constraints that are encountered while studying biomacromolecules using X-ray crystallography. We then describe some of the recent advances in experimental methods, both in crystallography and otherwise, to remedy some of the limitations. Next, we expound the crucial contribution of computational methods in complementing, as well as supplementing, experimental methods in order to study protein structural dynamics. Finally, we conclude the review with some of the limitations of computational methods and the future prospects.

## 2. Constraints in Crystallography for Studying Protein Structure and Dynamics

**Figure 1:**
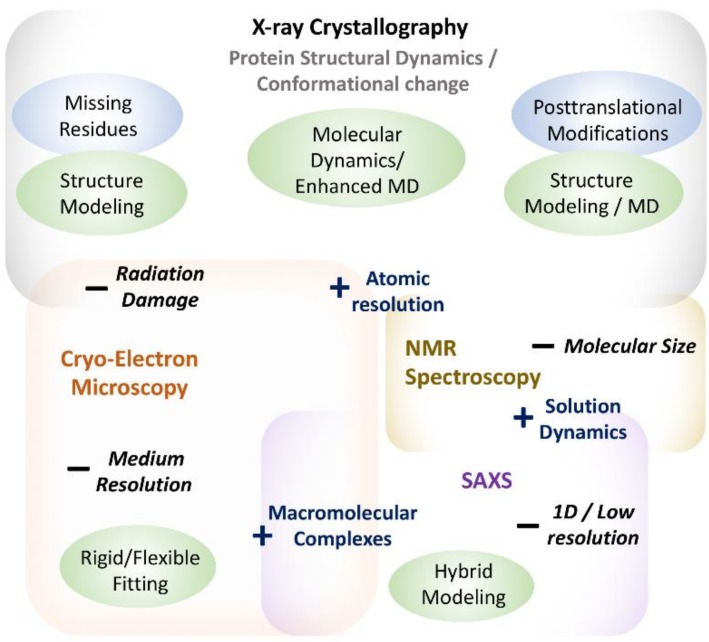
Summative figure showing strengths and limitations of various experimental methods in understanding protein structure and dynamics. MD stands for Molecular Dynamics. Different boxes represent different experimental methods with “+” sign showing their strengths and “−” sign showing their limitations. Computational methods addressing the limitations are shown in green ellipses.

X-ray crystallography has been the most important method for exploring the three-dimensional structure of biological macromolecules over time [1,2]. It is so far the most reliable and widely used method of obtaining atomic-resolution information about protein structure. Moreover, owing to well-established experimental protocols and data analysis methods, it remains a method of choice for the pharmaceutical industry to explore protein–drug interactions [2]. However, despite the various strengths of X-ray crystallography, it suffers from certain constraints (Figure 1), arising from a variety of different sources [6].

### 2.1. Challenges in Data Collection and Interpretation of Diffraction Data

One of the first challenges in studying protein structure using crystallography is obtaining good-quality protein crystals. Continued advances in the protocols and instrumentation allowed obtaining X-ray structures of larger systems at higher resolution; however, getting protein crystals that diffract to a high resolution remains a road block that protein crystallographers need to cross [7]. The protein crystallization process, being a multiparameter-dependent process, requires the tuning of several physical and biochemical factors for different proteins. The challenges include, but are not limited to, solubility of macromolecular complexes, membrane protein crystallization, disorder in the protein leading to unstable crystals, etc. The historical development, challenges, and future of protein crystallogenesis were previously reviewed in detail elsewhere [7].

Radiation damage to the protein crystals has long been a serious problem in biomacromolecular crystallography. Irradiation of crystals by X-rays causes the generation of free radicals that affect the overall diffraction intensity, and hence, the resolution and quality of structural model [8]. Apart from this overall reduction in quality, the damage caused by free radicals can cause specific changes in biomolecules, such as photoreduction, breaking of bonds, etc., particularly in the solvent-exposed regions of the proteins including active sites and cofactors bound to proteins, which can lead to incorrect conclusions [9]. Serial femtosecond crystallography, described in detail later, is a new approach for circumventing this issue. 

Furthermore, once the diffraction data are collected, the models created using the data might sometimes be suboptimal, particularly for medium- to low-resolution structures. Such uncertainty was explored extensively in protein–ligand systems, where it was found that the model for ligands is often overinterpreted [10,11]. 

Since the model of the structure generated using X-ray crystallography is determined as an averaged model from the diffraction data obtained from multiple copies of the protein in crystal, the structural heterogeneity and anisotropy in the atomic motions is usually lost, except for very-high-resolution structures [12]. Considering that the function of proteins may be dependent on the sub-angstrom-scale movement of atoms [13,14,15], ambiguity or inaccuracy in the crystal structure model might confound the understanding of the structure–function relationship.

### 2.2. Challenges in Retrieving Biochemical Information from Crystal Structures

#### 2.2.1. Crystal Environment Artefacts

Crystals comprise unit cells which are in turn composed of several asymmetric units. As a result of crystallization, the protein molecules in these asymmetric units or neighboring symmetry-related molecules interact, giving rise to spurious interactions that are absent in functional molecules in cells and can be referred to as crystal packing artefacts [16]. Despite using similar forces, crystal contacts are not considered to be strong interactions when compared to biologically relevant interactions [17,18,19]. However, these interactions might still lead to the stabilization of certain higher-energy conformations, as explored in λ Cro dimer structures using extensive molecular dynamics and replica-exchange molecular dynamics simulations [20,21]. It is sometimes observed that the same protein in different crystal forms takes different conformations, which suggests the role of crystal contacts in obtaining the given conformation [22,23].

Apart from crystal packing, the crystal chemical environment (buffer salt concentration, pH, etc.) can also affect the conformation observed in the crystal structure. In human protein kinase CK2, a high concentration of lyotropic salts in the crystallization buffer is known to support the closed conformation of the kinase, which was rarely observed in solution dynamics [24,25].

#### 2.2.2. Cryo-Cooling Effects

Cooling the crystals to a very low temperature (typically 100 K) for diffraction data collection—referred to as cryo-cooling—has been an indispensable technique in X-ray crystallography for about three decades now [26]; currently, most X-ray crystallographic structures are obtained using this technique. Cryo-cooling not only decreases the incidence of radiation damage due to high-energy X-ray radiation, but also decreases the overall atomic motion, thus improving the diffraction resolution [26]. However, the protein assumes distinct dynamics between the cryogenic temperature and the physiological temperature. The dynamics of proteins in crystals are described by B factors despite their limitation [27], and typically, the B factor increases at higher temperatures, with inflection near 180 K, which is associated with glass transition [28]. Heterogeneity in cooling across crystals also poses challenges in determining the effects of cryo-cooling on protein structure, as discussed in Section 3.1.3. [29]. 

#### 2.2.3. Missing Residues, High-Flexibility Regions

Crystal structures often contain multiple residues whose coordinates are missing from the model. As per a recent study, about 69% of PDB files were found to contain missing residues [30]. In particular, low-resolution structures (>3 Å) were found to be missing about 10% of the residues [30]. This is usually the result of high flexibility in the solvent-exposed loop regions of the proteins, which leads to highly ambiguous or no electron density for that region. These missing residues can make it difficult to understand the biological processes when they are at or near a functionally important region of the protein. 

Intrinsically disordered proteins (IDPs) or regions of structured proteins with intrinsically disordered regions form some of the toughest targets for structural and dynamics analysis [31]. The high flexibility of such proteins makes it almost impossible to get good-quality crystals, and hence, X-ray diffraction data. The structural elucidation of such IDPs also remains challenging using other experimental techniques.

#### 2.2.4. Missing Water Molecules and Solvent Information

Most biomolecules are surrounded by water within cells. Apart from playing crucial roles in regulating the physico-chemical properties of these molecules, water molecules also participate directly in biochemical reactions [32,33,34]. Usually, crystal structures at higher resolution have some ordered water molecules that show electron density, and hence, can be modeled. However, in most cases involving lower-resolution structures, the water molecules cannot be observed. Even at high resolution, the role of dynamic water molecules in function cannot be studied using crystallography.

Notwithstanding the challenges mentioned above, crystal structures determined so far provide crucial mechanistic insights into the biological phenomena under observation, and can be used to design further experimental and computational studies to fully characterize dynamics and functional mechanisms. Having described the limitations in obtaining structural and dynamic information using X-ray crystallography, we proceed to review the recent advances in experimental methods that contributed in overcoming these limitations.

## 3. Recent Experimental Advances for Enhancing/Complementing Crystallography

### 3.1. Advancements of Crystallography Methods

Several advances were made recently in the field of crystallography to address some of the limitations discussed above. Most of these advances were heralded by path-breaking developments in instrumentation and in experimental protocols.

The advent of increasingly high-energy X-ray sources, such as second- and third-generation synchrotrons, dramatically improved the resolution for biomacromolecular crystallography [35]. In addition, high-energy X-rays were also shown to cause less radiation damage, although there are conflicting reports about the relationship between the energy of X-rays used and radiation damage [9,36,37,38].

#### 3.1.1. Crystallographic Refinement Methods

As mentioned in Section 2.1, despite the presence of structural heterogeneity and dynamics in protein crystals, most crystal structures in the Protein Data Bank contain single conformational models [39]. Several computational methods were developed to model this heterogeneity present in the electron density maps [39,40,41,42,43]. Most of these methods involve sampling different conformations to improve the fit to electron density. One such method is *phenix.rosetta_refine*, which combines the crystallography refinement protocol of Phenix [44] and the conformational sampling by popular modeling software Rosetta to provide good models from low-resolution data [45]. Recently, a crystallographic refinement method was proposed by Pearce et al. [46] that makes use of multiple diffraction datasets to model regions with weak or ambiguous electron density [46]. Further development and the broad usage of such computational methods can lead to abstraction of more accurate models from crystallographic data.

#### 3.1.2. Serial Femtosecond Crystallography and Single-Particle Experiments with X-ray Free-Electron Laser (XFEL) Sources

The two most challenging methodological problems associated with crystallography are growing the crystals and radiation damage due to long-term exposure to high-energy radiation. The advent of X-ray free-electron lasers (XFELs) opened an avenue for solving both these problems [47,48]. The femtosecond-long XFEL pulses generate diffraction patterns before the destruction of protein crystals [49]. The high-energy pulse allows measurements from nanocrystals, thereby circumventing the need for growing large crystals. However, since the crystal is completely destroyed in a single encounter, a large number of diffraction measurements need to be collected using a stream of crystals [48]. This method holds enormous promise for determining the structures of conventionally challenging membrane proteins [50]. Femtosecond crystallography is also used to study the dynamics of proteins in time-resolved experiments [51,52,53,54], as was done with a synchrotron beam [55].

In addition to crystallographic studies, attempts began extracting the structural information of biological systems in near-native state using XFEL through single-particle analysis of non-crystalline samples [56,57,58]. Although, the experiments are currently performed for sub-μm systems and the resolution is quite limited, experimental techniques and computational algorithms are actively being developed toward the goal of structure analysis [59,60,61,62,63,64].

#### 3.1.3. Multi-Temperature/Room-Temperature Crystallography

The effects of cryo-cooling on protein crystal structures are not fully understood. Experimentally, Fraser and coworkers investigated these low-temperature effects extensively [65,66]. They suggested that cryo-cooling affects the side-chain conformational ensemble in a significant and biologically relevant manner [65,66]. In particular, a side-chain conformer, suggested to be catalytically important, was only detectable with room-temperature diffraction of cyclophilin A [65]. By comparing NMR experimental and crystal structures of dihydrofolate reductase, they also demonstrated that the room-temperature crystal structure is indispensable for investigating protein dynamics [67]. In addition, the cooling process may introduce further heterogeneity because of different timelines of cooling [29].

The recent development of XFEL also provides the opportunity for room-temperature crystallography. Sirerra et al. [68] studied the room-temperature structure of ribosome in comparison with cryo-cooled synchrotron data, showing a 3.6-Å shift of the h28 phosphate backbone. Such a large change can have a significant impact on the ligand binding affinity [68]. 

#### 3.1.4. Crystal Contact Free Space

Structures solved by X-ray crystallography capture only a snapshot or an averaged representation of a dynamic protein. However, these dynamic motions are essential for studying the biological function. Crystal contacts play a role in blocking these internal motions in a protein unless the dynamic region of the protein is located where it cannot interact with its neighboring molecule in the crystal. A new fusion protein method was developed for studying large dynamic motions within protein crystals by minimizing crystal contacts [69]. In this method, a target protein is fused with a tag protein via a rigid linker causing the formation of crystal contact free space (CCFS) in the protein crystal. The dynamic region is localized in the CCFS, and thus, is free from any crystal contact. This method was applied to a mitochondrial outer membrane protein, Tom20, which binds to presequence to import proteins into the mitochondria, and an archaeal oligosaccharyltransferase protein (AgIB) [69]. Although flexible regions free from crystal contacts result in weaker electron densities, using proper data0processing techniques and computational methods, dynamical information can be obtained.

### 3.2. Other Biophysical Techniques

In this section, we review some of the biophysical methods that give important information about protein structure and dynamics, which recently gained importance owing to improved experimental and computational protocols. As detailed analyses of each of these methods is beyond the scope of this review, we mention the strengths of each of the methods when compared to crystallography and cite recent reviews that provide details of these methods.

NMR spectroscopy is a powerful method for studying not only the structure and dynamics of biomacromolecules in solution, but also ligand binding [70], protein–protein interaction [71], and protein–nucleic-acid interaction [72]. Historically, one of the biggest limitations of studying biomacromolecules using NMR spectroscopy was its inability to deal with large molecules (>20 kDa). However, owing to recent advances in labeling techniques and new experimental protocols, structures of several protein complexes with sizes in the range of hundreds of kDa were determined [73,74]. Apart from this, NMR spectroscopy is currently being successfully utilized to understand the role of low-population conformations of proteins in solutions, which were previously impossible to investigate [75]. Further advancements, such as solid-state NMR spectroscopy, in combination with computational methods and scattering studies, enable the exploration of biological systems such as membrane proteins, which are challenging to study using crystallography [76,77].

Hydrogen exchange mass spectrometry (HX-MS) is a comparatively old technique for studying conformational changes in proteins that historically eluded scrutiny through crystallography or NMR spectroscopy. HX-MS quantifies the exchange of amide hydrogen with deuterium in solution and provides information about the tertiary structure and dynamics of the protein [78]. HX-MS is helpful in elucidating conformational changes in proteins upon ligand binding [79] and during allosteric regulation [80]. Recently, HX-MS was used to understand the conformational dynamics of DNA binding and Cas3 recruitment by the cascade complex [81].

Cross-linking mass spectrometry (XL-MS) is an important experimental technique for gaining insight into protein–protein interactions and structures of large macromolecular complexes. Recent years saw considerable development both in the cross-linking methodology and in data processing and visualization (recently reviewed by Leitner et al. [82]). XL-MS usually involves creating covalent bonds between spatially close regions of protein complexes, followed by proteolysis and subsequent mass spectrometry to determine the regions enriched in cross-linked peptides. The data obtained from XL-MS can be used to generate distance restraints and were recently used extensively to model large macromolecular complexes (See Section 4.2). 

Transmission electron microscopy has been used to observe biological systems on the scale of micrometers to tens of nanometers for a long time. However, owing to recent advances in instrumentation, including better electron guns, electron detectors, automated mounting systems, and image-processing software and algorithms [83], there was a resolution revolution in the field of cryo-electron microscopy (cryo-EM) [84] with volume maps reaching resolutions of up to 2 Å. This made several difficult crystallographic target protein complexes accessible to structural and dynamical analysis [85,86,87,88].

Small-angle X-ray scattering (SAXS) is a powerful and well-established method for studying proteins in solution. It can provide several important parameters about the protein in its native state, including molecular weight, overall shape, and radius of gyration [89]. With recent advancements in synchrotron radiation sources, there was a resurgence in the use of SAXS for studying biomolecular structure and dynamics [90,91]. It is particularly helpful in determining low-resolution shape information about the oligomeric states of proteins [92], as well as the structure and dynamics of intrinsically disordered proteins [93,94] and integral membrane proteins difficult to study using other biophysical techniques [95].

As described in the previous section, there was immense advancement in experimental methods for studying protein structure and dynamics. However, each method has its own limitations, and computational methods became essential in mitigating these limitations, integrating results from multiple experimental sources and providing a better understanding of protein structure and dynamics. In the following section, we review the computational methods that are crucial in the exploration of protein structure and dynamics.

## 4. Computational Methods for Complementing/Supplementing Experiments

### 4.1. Protein Structural Modeling

The inability to obtain a protein of interest in considerable quantities, either via expression or purification, still remains one of the first challenges in studying the protein–structure–function relationship. For all such cases where protein structure determination proves challenging, structure prediction remains one of the most sought-after computational methods. With an ever increasing number of known protein structures and the development of better algorithms, there has been a considerable improvement in the quality of model predictions [96]. 

The most accurate and popular methods of protein structure prediction are still dependent on the availability of the structure for a homologous protein. Homology- or template-based modeling methods involve using the structural information from a sequentially similar region of a homologous protein for which the structure is already known. Template-based methods improved considerably over the years, primarily due to an increase in the number of proteins for which structures are known and due to improvements in sequence-alignment methods [97].

Despite the impressive increase in the number of experimentally determined protein structures, there is still a large gap between sequence space and structure space. Consequently, for a large number of proteins, templates are not available to assist in structure prediction. To understand the structure for such proteins, several ab initio or template-free methods were developed. Although such prediction methods are severely limited with regards to the length of the protein sequence, certain recent advances in reliable residue–residue contact predictions considerably improved their accuracy [96,98].

Apart from playing a crucial role in understanding the structure and function of proteins difficult to study using experiments, protein structural modeling is routinely used to study regions that are missing from the crystal structures. Modeling the regions of missing coordinates in protein crystal structures forms one of the first steps in protein structural and dynamics studies using varying computational methods.

### 4.2. Hybrid Methods for Studying Protein Complexes

As discussed in Section 3, there was immense advancement in experimental methods that provide low-resolution or complementary information about the overall structure and dynamics of proteins and their complexes in solution. Simultaneously, computational methods were developed to integrate such information coming from multiple sources, with existing high-resolution crystal and NMR structural data for gaining insight into solution structure and dynamics. Hybrid methods, utilizing information from multiple experimental sources and integrating them using computational algorithms, are particularly successful in understanding the structure and dynamics of large macromolecular complexes [99,100,101,102,103,104]. 

Volume maps obtained even from state-of-the-art cryo-EM experiments typically range around 3–4 Å resolution, which is insufficient to obtain atomic level information. Crystallography data, although high resolution, might not represent a functional or solution state. Computational methods integrating information obtained from these two complementary sources can, thus, supplement the overall information and provide novel insight into both large-scale, as well as atomic-level, structure and dynamics. Several methods were developed for fitting high-resolution crystal structures to EM density maps (for a recent review, see Kim et al. [105]). These methods rely on different approaches to deform coordinates of known high-resolution crystals or NMR structures to fit low-resolution EM maps, such as normal-mode-based flexible fitting [106,107], coarse-grained model-based flexible fitting [108,109], all-atom molecular-dynamics-based fitting [110], deformable elastic-network-based fitting [111], Monte Carlo method-based fitting [112], or a combination of these methods [113].

SAXS provides information about the overall shape of large macromolecule in solution. This is very helpful in understanding the large conformational changes associated with the function of macromolecules. The low-resolution information obtained from SAXS can be used to reconstruct high-resolution structural models using existing crystal structures [114,115,116,117]. Cross-linking mass spectrometry data provide spatial restraints between different components of large macromolecular complexes. These restraints are utilized by integrative modeling software to elucidate the structure and dynamics of these complexes [118,119,120,121,122]. Hybrid methodologies were also developed recently to utilize X-ray free-electron laser diffraction data to understand protein structure and dynamics [123].

### 4.3. Molecular Mechanics Methods

**Figure 2:**
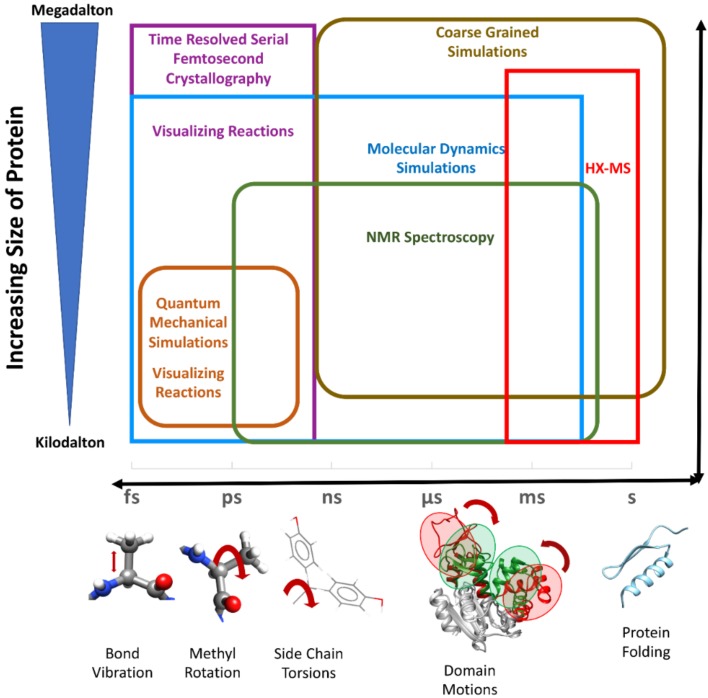
Protein dynamics can be modeled at several length and time scales. Various experimental and computational methods (shown in same colored text and boxes) are used to understand the multiscale dynamics of proteins and their complexes. Red arrows show different motions in the molecules happening at femtosecond (fs), picosecond (ps), nanosecond (ns), microsecond (μs) and millisecond (ms) time scale. Protein folding can have a time scale of seconds (s) to minutes or even hours.

Biomacromolecules (Proteins, DNA, and RNA) of interest can be described at multiple scales of detail. At the small scale, the electron distribution of each atom in the molecule is described, and the dynamics of these atoms are studied using quantum mechanics. Although this approach is extremely useful and accurate in studying small molecules and mapping reactions in localized active-site pockets of enzymes [124], it becomes computationally infeasible for large systems involving thousands of atoms. At a lesser detailed level, the atoms of the biomacromolecules themselves are modeled as particles, and the forces between these particles are described and calculated based on semi-empirically-derived force-field potentials based on classical mechanics (Figure 2). The methods using such descriptions, referred to as molecular mechanics methods, form a quintessential part of computational simulations of biomacromolecules [125]. Molecular dynamics (MD) simulations help study the time evolution of dynamics of a molecular system by integrating the forces acting on each atom of the system at successive time points. Since the first MD simulations of colliding hard spheres [126], the method was developed immensely and is used extensively to study biological processes of varying length and time scales [125]. The emergence of faster processors, particularly graphics processing units, and the development of faster algorithms to perform calculations on these processors, led to a revolution in large-scale studies using MD simulations. This led to the structural and dynamic exploration of large macromolecular machines at atomistic resolution in unprecedented detail.

#### 4.3.1. Coarse-Grained Modeling

Despite breakthroughs in hardware and software capabilities of modern computer systems, it is still a challenging task to study protein dynamics in the time range relevant for biological functions, especially for important macromolecules. In order to address this, simplified coarse-grained (CG) models were developed and are used successfully for the last four decades. CG models are simplified representations of biological macromolecules, often treating amino-acid residues, secondary structural elements, or whole domains as single entities. This leads to a dramatic reduction in the degrees of freedom, consequently making these methods computationally much less expensive than molecular dynamics simulations. Structure-based models like Go models that assume a smooth funnel-shaped energy landscape are particularly useful in studying the folding of proteins [127,128]. Inspired by the seminal work of Monique Tirion [129], elastic network models form another class of coarse-grained model that contributed to understanding protein dynamics in diverse biological systems [130,131,132,133]. Network-based coarse-graining of protein structures is also able to provide insight into protein folding [134] and allosteric communication [134,135,136].

#### 4.3.2. Enhanced MD Simulations

The rugged conformational landscape of proteins makes conventional MD simulations vulnerable to be trapped in one of the local, non-functional free-energy minima [137]. In order to overcome this problem, several enhanced MD methods were developed [137]. As the range is broad, we can only review a limited number of methods. For more detail, readers are referred to extensive reviews by Bernardi et al. [137], Mitsutake et al. [138], and Valsson et al. [139]. 

Methods such as replica exchange [140], umbrella sampling [141], and multicanonical algorithms [142,143] utilize equilibrium sampling of non-Boltzmann weights, which can enhance sampling efficiency. The information of canonical ensembles can be obtained via proper post-processes. The replica-exchange method is especially suitable for parallel computing, and its post-process is straightforward. Hence, the MD version of the replica-exchange method, known as replica-exchange molecular dynamics (REMD) [140] is widely used in biomolecular systems. REMD and its related methods have interesting applications, including studying pH dependence of biomolecules [144], drug design [145], cold denaturation [146], and biological systems with strong phase transitions [147]. Furthermore, recent theoretical progress enabled the extraction of kinetic rates using these methods [148]. 

Some other methods use non/quasi-equilibrium sampling. In steered MD [149], a molecule or a part of it is dragged with an artificial external force so that desired conformational changes can be studied. Even with such an external force, it is possible to extract free energy via Jarzynski equality [149]. The Wang–Landau method [150] and metadynamics [139,151] employ dynamically updated potentials or weights of sampling so that the system can sample a wider range of energy or reaction coordinates which are specified a priori. 

### 4.4. Crystal MD Simulations

Crystal structures typically portray a single averaged model of protein structure, which loses information regarding the dynamics of the protein not only in solution, but also in the crystal itself. In terms of exploring structural heterogeneity within the protein crystal, there were a few recent attempts to include the ensemble view of the structure in crystal model building. (See Section 4.2). However, molecular dynamics simulations have been used to understand the structural dynamics in the crystal environment for a long time [152,153]. 

In these simulations, the entire unit cell is simulated in an environment very similar to the experiment. This makes the comparison between X-ray data and simulations rather informative; however, these are computationally expensive simulations as compared to conventional MD simulations, owing to larger starting structures and delayed convergence [154]. Nevertheless, crystal MD simulations provided novel insights into the dynamics of protein and solvent in crystal [155], the energetics of the packing interface [21], crystal packing effects [156,157], protein–protein and protein–detergent interactions in crystals of membrane proteins [158], solute diffusion in protein crystals [159], and the behavior of water and ions in protein crystals [160].

## 5. Novel Insights from Computational Methods

The crucial role of computational methods in understanding protein structural dynamics cannot be overstated. In the following sections, we review novel insights gained using computational methods, either independently or in concert with experiments.

### 5.1. Exploring the Structure and Dynamics of Large Macromolecular Complexes

Biological systems are complex and, often, a multidisciplinary approach is required to understand these complex systems. This is particularly true when it comes to understanding the structure–function–dynamics of large macromolecular machines within cells like ribosomes, proteasomes, spliceosomes, transcription initiation complexes, etc. Until recently, the only experimental method that provided high-resolution information of these macromolecular assemblies was X-ray crystallography. Despite great achievements in determining crystal structures of some of these complexes, the information for most of macromolecular complexes is missing. Recent phenomenal advances in the cryo-EM led to enormous breakthroughs in the understanding of macromolecular structures and dynamics [84].

The ribosome is a macromolecular machine responsible for the synthesis of all proteins inside cells. Owing to its crucial role in all living systems, structure–function studies of the ribosome have gone on for decades, and the determination of an atomic-resolution crystal structure of the ribosome enhanced the understanding of one of the most fundamental biological process [161]. However, the ribosome is a highly dynamic macromolecular complex that undergoes several conformational changes during protein translation [162]. Static structures obtained from crystallography and cryo-EM often fail to capture the transient dynamics between different states of this complex. Whitford et al. applied a hybrid approach of using a flexible fitting program, MDfit, along with experimental information from multiple sources, including X-ray crystallography, cryo-EM, and biochemical experiments, to shed light on the transient conformations involving transfer RNA (tRNA) translocation into the ribosome during the process of translation [163]. Additionally, despite the large size of the ribosome, considerable progress was made toward the molecular simulations of whole ribosomes. This was recently reviewed by Bock et al. [164]. 

The C-terminal domain (CTD) of RNA polymerase II comprises several repeats and is structurally disordered, leaving it inaccessible to most structure determination methods, including X-ray crystallography and cryo-EM. Consequently, despite its important role involving multiple protein–protein interactions during transcription, there was limited structural information detailing the CTD in complex with its cognate binding partners. Using integrative structural modeling, Jasnovidova et al. [102] determined the molecular architecture of the C-terminal domain of RNA polymerase with termination factor Rtt103. This involved assimilating existing and novel structural and other experimental data into a computational algorithm to determine the overall molecular architecture [102].

The conformational dynamics of the spliceosome, a megadalton-sized machine that processes precursor messenger RNAs to construct coding regions (exons), was studied at atomic resolution to delineate functional motions that were missing from the static structures [165]. Computational modeling and simulation also enabled the study of previously unexplored areas, such as describing the crowding effects inside the cytoplasm [166].

Going a step further on the length scale, hybrid methods involving cryo-EM, X-ray crystallography, molecular modeling, and molecular dynamics simulations were used to determine the structure and dynamics of a whole viral capsid comprising millions of atoms [167,168]. These studies provided crucial insights into the maturation and assembly process of the viral capsid.

### 5.2. Structural and Dynamical Effects of Post-Translational Modifications

There are hundreds of types of post-translational modifications (PTMs) of proteins that were found to be biologically important. Post-translational modifications like phosphorylation, methylation, acetylation, glycosylation, etc. were shown to play major roles in almost all biological processes [169]. These modifications cause changes in the structure, as well as the dynamics, of the proteins, modulating the functions of these proteins and their interacting partners [170]. 

In view of the importance of PTMs in the biological processes, the experimentally derived structural information for such modifications is considerably low [171]. Molecular modeling and simulations contributed significantly in understanding the role of PTMs in the structure and dynamics of proteins. Molecular dynamics simulations were used to study the effect of phosphorylation on the polar properties of protein surfaces, which could be crucial in understanding the interaction of proteins with other molecules [172]. In another molecular dynamics study, the role of *S*-glutathionylation of a particular cysteine residue in *Arabidopsis thaliana* BRASSINOSTEROID INSENSITIVE 1-associated receptor kinase (BAK1) was explored, and the effect of this PTM on the conformational dynamics reconciled with previous experimental observations [173]. 

### 5.3. Structure-Based Drug Design

Computational methods have contributed to almost every stage of drug discovery for a long time now [174]. Structure-based drug design conventionally focuses on high-throughput virtual screening using crystal structures of the target proteins; however, the role of dynamics in protein–drug interactions is being increasingly realized [175]. At the stage of initial virtual screening, instead of a static crystal structure, using an ensemble of structures for finding a lead molecule takes into consideration the inherent solution dynamics of the target binding site [176,177]. 

Virtual screening entails searching through millions of compounds for their ability to bind to the target protein. Pharmacophore-based screening simplifies this task by extracting a number of electronic and chemical features from a known ligand that can be used to screen for compounds with similar features. An intuitive extension of this approach is three-dimensional pharmacophore-based screening, where not only the chemical features but also the steric features are considered based on available protein–drug structures [178]. Conventionally, static single crystal structures were used to extract such pharmacophores; however, recently, molecular dynamics simulations of protein–ligand complexes were performed and used to generate dynamic pharmacophore models [179,180]. Using multiple crystal structures or conformations obtained from MD simulations was shown to reveal certain features that were missed when considering only single structures [181,182].

### 5.4. Understanding the Role of Water in Protein Structure and Function

Computational methods, particularly molecular dynamics simulations and quantum mechanics simulations, play crucial roles in understanding the role of water in the structure and function of biomacromolecules. Molecular dynamics simulations were used to understand the role of water molecules in protein–DNA binding [183], enthalpy–entropy compensation during protein–ligand interactions [184], proton transfer reactions in channel rhodopsins [185], etc. Furthermore, MD simulations were used to complement experimental methods like terahertz absorption spectroscopy [186], neutron scattering [187], and time-resolved fluorescence spectroscopy [34] to understand the structure and dynamics of folded and intrinsically disordered proteins.

## 6. Limitations of Computational Methods

Computational methods for studying protein structural dynamics were instrumental in understanding numerous biological processes and successfully shed light on phenomena that were inaccessible to experimental methods. However, like any other method, they also have certain limitations. In the following sections, we discuss some of the common limitations of the previously discussed computational methods.

### 6.1. Force Fields

Results obtained from any computational method are only as reliable and accurate as the parameters used in the development of that method. Molecular dynamics simulations use a combination of functional forms defining the interactions between different particles in the system and a set of predetermined parameters that are used to calculate forces between the particles [188]. Such force fields are constantly evolving with improved parameters to reconcile experimental observations and simulation results [189]. However, there are some key limitations to what can be studied using these force fields.

The force fields used to study solution dynamics of proteins were developed to model the behavior of folded proteins. This led to certain insufficiencies in studying intrinsically disordered proteins (IDPs) using the same force fields. Although simple modifications in some parameters of the force field improve the agreement between simulations and experiments, a more thorough evaluation and parameterization of forcefields is required for IDPs [190,191]. 

Most of the current force fields use fixed partial charges on the atoms to calculate electrostatic forces. Although these force fields were conventionally successful in simulating the behavior of molecules in homogeneous environments, their performance is limited in conditions where there are variations in local electric fields, and continued efforts are being made to resolve these limitations [192].

### 6.2. Sampling

While the sampling issues were addressed by several methodological developments as described above, the time scale of MD simulations is ultimately limited by the computational speed. Apart from the MD-specific machine Anton, which can reach sub-millisecond time scales per day [193], commonly used MD software packages that are used with general-purpose personal computer (PC/PC) clusters reach only sub-microsecond time scales per day, while using hundreds of central processing units (CPUs) [194]. Thus, the microsecond time scale is a practical limit for a single trajectory in common research groups as of 2018, although it is possible to replicate a system in question to increase statistics. The Markov state model [195] is one promising approach for inferring longer-time dynamics from limited MD trajectories, and any new framework that can work within such restraints should be useful and will open new gates for dynamics studies of protein. 

## 7. Summary and Future Directions

X-ray crystallography has long been the de facto method of choice to study protein structure and function. Despite several challenges and limitations, it continues to remain one of the most crucial sources of structural information. However, the realization of the importance of dynamics and the concomitant improvement in the accuracy and applicability of computational methods to be able to study the functional dynamics of proteins in native-like environments heralded the era of computation-driven structural biology. This was equally matched by the rise of novel experimental methods to elucidate the dynamics of proteins in solution at varying length and time scales. Considering the complexity of biological processes, it is now abundantly clear that a multipronged approach comprising inputs from several different experimental and computational methods is required to understand the important questions. Consequently, hybrid/integrative methods are starting to become the new norm in biomolecular structure and dynamics studies. This is evident from the fact that the Protein Data Bank now has a new field of classification named “hybrid methods”, consisting of structures determined using more than one kind of experimental method. Also, a wwPDB hybrid/integrative methods task force was established in order to formalize the representation, validation, archiving, and publication of structural and dynamics data generated using hybrid or integrative methods [196,197]. Computational methods form an essential part of such structure determination protocols as they enable combining and complementing data from multiple sources, and they will continue to play a crucial role in the field of structural biology with more advanced sampling methods, better supercomputers, and more experimental advances.
